# Implementation of music in the perioperative standard care of colorectal surgery (IMPROVE study)

**DOI:** 10.1111/codi.17200

**Published:** 2024-10-09

**Authors:** Ellaha Kakar, Oddeke van Ruler, Bas Hoogteijling, Eelco J. R. de Graaf, Erwin Ista, Johan F. Lange, Johannes Jeekel, Markus Klimek

**Affiliations:** ^1^ Department of Surgery and Intensive Care Unit Erasmus Medical Centre Rotterdam The Netherlands; ^2^ Department of Surgery IJsselland Hospital Capelle aan den IJssel The Netherlands; ^3^ Department of Anaesthesiology IJsselland Hospital Capelle aan den IJssel The Netherlands; ^4^ Department of Internal Medicine, Section Nursing Science Erasmus Medical Centre Rotterdam The Netherlands; ^5^ Department of Neonatal and Paediatric Surgery Intensive Care, Division of Paediatric Intensive Care Erasmus Medical Centre–Sophia Children's Hospital Rotterdam The Netherlands; ^6^ Department of Surgery Erasmus Medical Centre Rotterdam The Netherlands; ^7^ Department of Neuroscience Erasmus Medical Centre Rotterdam The Netherlands; ^8^ Department of Anaesthesiology Erasmus Medical Centre Rotterdam The Netherlands

**Keywords:** implementation, music, surgery

## Abstract

**Aim:**

Patients undergoing surgery experience perioperative anxiety and pain. Music has been shown to reduce perioperative anxiety, pain and medication requirement. This study assessed the feasibility and effectiveness of implementing a perioperative music intervention.

**Method:**

A prospective pre‐ and post‐implementation pilot study was conducted to assess adherence to the intervention and the initial effect of music on postoperative pain scores (Numerical Rating Scale, 0–10) compared to a control group. Secondary outcomes encompassed pain scores throughout hospital admission, anxiety levels, medication usage, complications and hospital stay length.

**Results:**

Adherence to the music intervention was preoperative 95.2%, intraoperative 95.7%, postoperative 31.9% and overall 29.7%. The intervention did influence postoperative pain. Patient's willingness to receive music was high (73%), they appreciated the intervention (median 8.0, interquartile range 7.0–9.0) and healthcare professionals were willing to apply the intervention. Music significantly reduced postoperative anxiety (2.0 vs. 3.0, *p* = 0.02) and the consumption of benzodiazepines on the first postoperative day (number of patients: zero [0%] vs. five [10%], *p* = 0.04).

**Conclusion:**

Implementation of music resulted in reduced postoperative anxiety and decreased consumption of benzodiazepines, and the strategy was feasible, but adjustments are needed to improve postoperative adherence. Both patients and healthcare professionals had a positive attitude towards the intervention.

**What does this paper add to the literature?**

Effective implementation of music in perioperative colorectal standard care may lead to reduced postoperative anxiety and benzodiazepine and opioid consumption. Music is easily applicable, presents no known risks, and can be considered as a component of standard perioperative care.

## INTRODUCTION

Patients undergoing surgical procedures often experience anxiety, pain and stress, experiences that may lead to complications, delayed postoperative recovery and extended hospitalization, despite protocolized management of pain [1, 2, 3, 4, 5, 6, 7, 8, 9, 10]. Preoperative anxiety can contribute to higher postoperative pain levels [11]. Patients with inflammatory bowel disease or colorectal cancer have higher anxiety rates compared to the general population [12, 13, 14]. Many of these patients undergo major surgical procedures, which add to their psychological stress [15, 16]. Recent studies have demonstrated significant positive effects of listening to recorded music on preoperative anxiety, postoperative pain, intraoperative sedative and postoperative analgesic requirements, as well as postoperative neurohormonal stress reactions [17, 18, 19, 20, 21].

Pharmacological agents such as benzodiazepines and opioids are widely used to ameliorate anxiety and pain, despite well‐known harmful side effects of pharmacological agents [22, 23, 24, 25, 26, 29, 30]. Preoperative benzodiazepine administration aimed at reducing anxiety has been associated with worse postoperative recovery and a higher risk for delirium [6, 27, 28]. In contrast, music listening is a non‐pharmaceutical intervention that is relatively inexpensive, easily applicable and without known side effects.

Efforts to implement music interventions in standard perioperative care have until now achieved adherence rates of only 36%–53% [31, 32, 33], which are considered too low to establish significant effects of the intervention. The explanation for the low adherence rates may be attributed to the failure to address potential barriers before implementation. The IMPROVE (Implementation of Music intervention in colorectal PeRiOperatiVe standard carE) study was set up to achieve a tailored implementation of music interventions in a validated manner [34]. The implementation study reported here aimed to assess (a) adherence to the music intervention [35] and (b) the impact of the implemented music on pain scores on the first postoperative day (POD).

## METHOD

**FIGURE 1:**
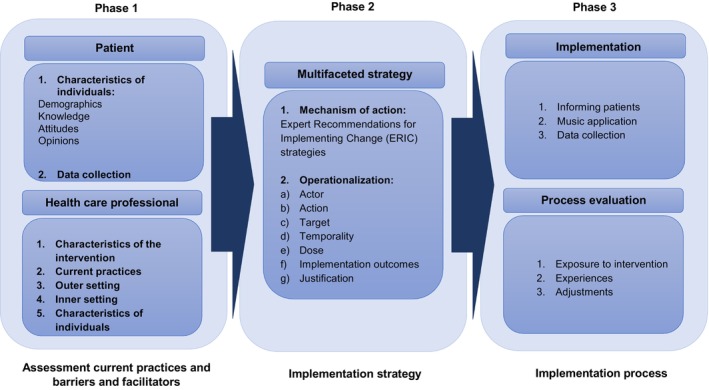
Overview of study phases.

This prospective, single‐centre pilot implementation study was conducted at a regional hospital (IJsselland Hospital) in the Netherlands from 11 December 2019 to 24 February 2022. The Medical Ethics Review Board of the Erasmus University Medical Centre had approved the study protocol (registration number MEC‐2019‐0563). The study was registered in the National Trial Register (www.clinicaltrialregister.nl, ID NL8071), and the protocol has been published previously [34]. Implementation of music intervention adhered to the Consolidated Framework for Implementation Research (CFIR) [36, 37]. In line with the CFIR recommendations, the study was organized into three phases:

- Phase 1 (pre‐implementation): Identification of facilitators and barriers for implementing recorded music in the elective colorectal surgery standard of care procedure
- Phase 2: Development of a strategy to implement recorded music interventions in the perioperative standard of care procedure of elective colorectal surgery in the IJsselland Hospital based on the results of phase 1, as described in a previously published paper [38]
- Phase 3 (post‐implementation): Application of the tailored implementation strategy and assessment of adherence to the music intervention by patients and healthcare professionals during perioperative care; additionally, assessment of the effect of the implemented music intervention on clinical outcomes (see Figure 1), as described in the current paper

### Participants

Patients who had undergone (pre‐implementation phase) or were to undergo (post‐implementation phase) a surgical procedure for inflammatory bowel disease or colorectal cancer, as well as general surgeons, anaesthesiologists, gastro‐intestinal surgery nursing ward and anaesthesiology nurses involved in the perioperative process were eligible. Exclusion criteria for patients comprised severe hearing impairments interfering with verbal communication, an inability or unwillingness to receive the intervention, and inadequate command of both Dutch and English.

### Outcomes

The primary aims included assessing the adherence (expressed as a percentage) of both healthcare professionals and patients to applying the music intervention in elective colorectal surgical procedures, and to assess the initial impact of the applied music intervention on the first POD pain scores, evaluated using the Numerical Rating Scale (range 0–10). Neither healthcare professionals nor patients were blinded to the intervention. Adherence was measured across preoperative, intraoperative and postoperative phases. Secondary objectives involved evaluating patient and healthcare professional attitudes/experiences via questionnaires, patient willingness for intervention, music's impact on postoperative pain/anxiety throughout hospitalization, preoperative and postoperative anxiety levels on POD 1, analgesic medication use, postoperative complications (Clavien–Dindo score) [39, 40], and hospital and intensive care unit length of stay (LOS). Opioid consumption was reported as milligrams of morphine equivalents (morphine intravenous [iv] [mg] + fentanyl iv [μg]/10 + remifentanil iv [μg]/10 + sufentanil iv [μg], piritramide/1.5 + oxycodone/1.5) [33]. Benzodiazepine consumption was reported as lorazepam equivalents (lorazepam + temazepam/10 [41] + oxazepam/15 [41] + diazepam/5 [42] + bromazepam/5 [43] + zopiclon/3.75 [42]). Successful implementation was defined as adherence to the music intervention by more than 70% of both patients and healthcare professionals [35, 44, 45].

### Implementation strategy (phase 2)

Data S2 shows the complete implementation strategy, based on the barriers and facilitators assessed in phase 1 [38] and selected by two members of the research team (EK/EI). The approach to addressing barriers was based on the Expert Recommendations for Implementing Change strategies [46], and it was operationalized using the seven dimensions proposed by Proctor et al. [47, 48]. The complete strategy development has been previously published in a protocol paper [34].

### Implementation process

**FIGURE 2:**
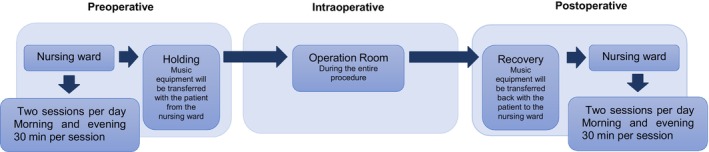
Planned intervention implementation.

The implementation strategy aimed to provide patients their own choice of music, twice daily, 30‐min recorded music sessions via headphones throughout their hospital stay (Figure 2). This duration was chosen based on literature indicating its clinical relevance. Patients were informed about these interventions using methods established in phase 1, and the timing of this information was optimized during that phase. Patients provided written consent for data collection from electronic medical records for primary and secondary outcomes. Implementation began on 1 April 2021 and lasted 6 months.

### Process evaluation

During the implementation phase, the execution of the strategy was continually reviewed and evaluated through the following steps.

1. The coordinating researcher (EK) daily contacted the nursing ward to assess the extent to which the intervention was offered to patients scheduled for surgery.
2. On the first day following a patient's discharge from the hospital, adherence to the implementation strategy was evaluated by phone using a self‐made questionnaire (Data S1).
3. A member of the research team evaluated the adherence to the intervention each month throughout the implementation phase.
4. The implementation strategy underwent regular evaluation by a dedicated team comprising professionals from each healthcare group involved in the process (as described in phase 1 [38]). These evaluations, informed by feedback from patients and the nursing team, aimed to assess if the intended implementation was successful and to gauge the experiences of those exposed to the intervention, including patients and hospital staff.
5. Based on these process evaluations, the implementation strategy was adjusted where necessary.
6. The healthcare professional questionnaire, used in phase 1, was adapted and administered again at the end of the implementation process (phase 3) to re‐assess knowledge, attitudes and perceptions.

### Statistical analysis

Adherence is represented as a percentage (Figure 2). Data from pre‐ and post‐implementation, phase 1 and phase 3 respectively, were used to evaluate the initial impact of music intervention on POD 1 pain scores, since its assessment is more generalized. Pain management for colorectal surgery in the IJsselland Hospital encompasses patient‐controlled analgesia for laparoscopic surgery and epidural analgesia for primary laparotomy procedures or procedures with a high conversion chance. A minimum reduction of 12 mm on the Visual Analogue Scale (VAS) for pain has been shown as clinically relevant [51]. To measure the impact of music intervention post‐implementation on postoperative pain we performed a sample size calculation based on reported mean pain scores and standard deviation after colorectal surgery in the papers by Kaminski et al. [49] and Mouawad et al. [50] considering that pain scores are equivalent to our patient population since scores are based on a large sample and the surgical procedure and pain management are similar. We used the mean pain scores measured with the VAS of patients on the first POD using patient‐controlled analgesia (*n* = 173, VAS = 4.6 ± 2.0) which yielded the largest sample size. We aimed to obtain a power of 80%, with level of significance set at 5% (*p* = 0.05), planned two‐sided testing and a dropout rate of 10%. Consequently, the sample size was 50 patients pre‐implementation and more than 50 patients post‐implementation [34].

The normality of the data was assessed using the Shapiro–Wilk test and Q–Q plots for graphical confirmation. Differences in secondary outcomes between the pre‐ and post‐implementation groups were analysed using parametric tests (Student's *t* test and chi‐squared/Fisher's exact tests) for normally distributed data, and results are presented as mean (standard deviation). Non‐parametric tests (Mann–Whitney *U* test and chi‐squared/Fisher's exact tests) were used for data with non‐normal distribution, and results are presented as median (interquartile range, IQR). A multilevel linear regression with random intercepts was applied to compare the change in pain scores over time (Δ) between the two groups using random effects [52]. A mixed linear regression model was employed to correct for baseline differences. The significance level for all tests was set at <0.05.

## RESULTS

### Patient demographics

Demographic characteristics are presented in Table 1. The post‐implementation patients had a slightly higher proportion of women (52.0%). Most of the patients underwent a laparoscopic procedure (79.0%) for segmental colectomy (37.0%) or rectum resection (22.0%). In the post‐implementation music group, there were significantly more men than in the pre‐implementation control group (58.0% vs. 38.0%, *p* < 0.05).

**TABLE 1:** Demographic characteristics of the patients.

	*N*	Overall	*N*	Pre‐implementation	*N*	Post‐implementation	*p*
Age, years, median (IQR)	100	62.5 (53.8–72.0)	50	52.5 (49.8–71.8)	50	63.0 (56.0–73.0)	0.50
Male, %	48	48.0	19	38.0	29	58.0	<0.05
BMI	100	25.6 (23.3–29.0)	50	25.7 (23.1–29.3)	50	25.4 (23.8–28.9)	0.91
Weight	100	79.7 (68.8–89.9)	50	77.5 (67.4–86.0)	50	80.7 (69.8–90.4)	0.59
Diagnosis, %							
Cancer	70	70.0	32	64.0	38	76.0	0.61
Crohn's disease	16	16.0	10	20.0	6	12.0	
Ulcerative colitis	7	7.0	4	8.0	3	6.0	
Other^a^	7	7.0	4	8.0	3	6.0	
Type of surgery, %							
Colectomy	37	37.0	23	46.0	14	28.0	0.11
Rectum resection	27	27.0	11	22.0	16	32.0	
Sigmoid resection	16	16.0	4	8.0	12	24.0	
Ileocaecal resection	11	11.0	6	12.0	5	10.0	
Colostomy	5	5.0	4	8.0	1	2.0	
Other	4	4.0	2	4.0	2	4.0	
Technique, %							
Laparoscopic	79	79.0	41	82.0	38	76.0	0.14
Laparotomy	7	7.0	1	2.0	6	12.0	
Conversion	14	14.0	8	16.0	6	12.0	
ASA score, %	99		50		49		0.16
1	5	5.1	2	4.0	3	6.1	
2	53	53.5	22	44.0	31	63.3	
3	40	40.4	25	50.0	15	30.6	
4	1	1.0	1	2.0	0	0.0	
Epidural, %	13	13.0	6	12.0	7	14.0	1.00
History, %							
Oncological^b^	71	71.0	33	66.0	38	76.0	0.27
First time surgery	38	38.0	17	24.0	21	42.0	0.41
Chronic pain^c^	11	11.0	5	10.0	6	12.0	1.00
Psychiatric^d^	9	9.0	5	10.0	4	8.0	1.00

Abbreviations: ADD, attention deficit disorder; ADHD, attention deficit hyperactivity disorder; ASA, American Society of Anesthesiologists; BMI, body mass index; CTS, carpal tunnel syndrome; HNP, hernia nuclei pulposi; IQR, interquartile range; *N*, number of patients; PNP, polyneuropathy.

^a^ Other: constipation due to Sjogren's disease, diverticulitis/diverticulosis, atonal colon due to Ehlers–Danlos syndrome, obstructive defaecation syndrome.

^b^ Oncological history: including current oncological problem.

^c^ Chronic pain history: HNP, CTS, migraine, neuropathic pain, PNP.

^d^ Psychiatric history: depression, ADHD, ADD, bipolar disorder, autism, delirium, Parkinson's disease, burn‐out, claustrophobia.

### Adherence to perioperative music

**FIGURE 3:**
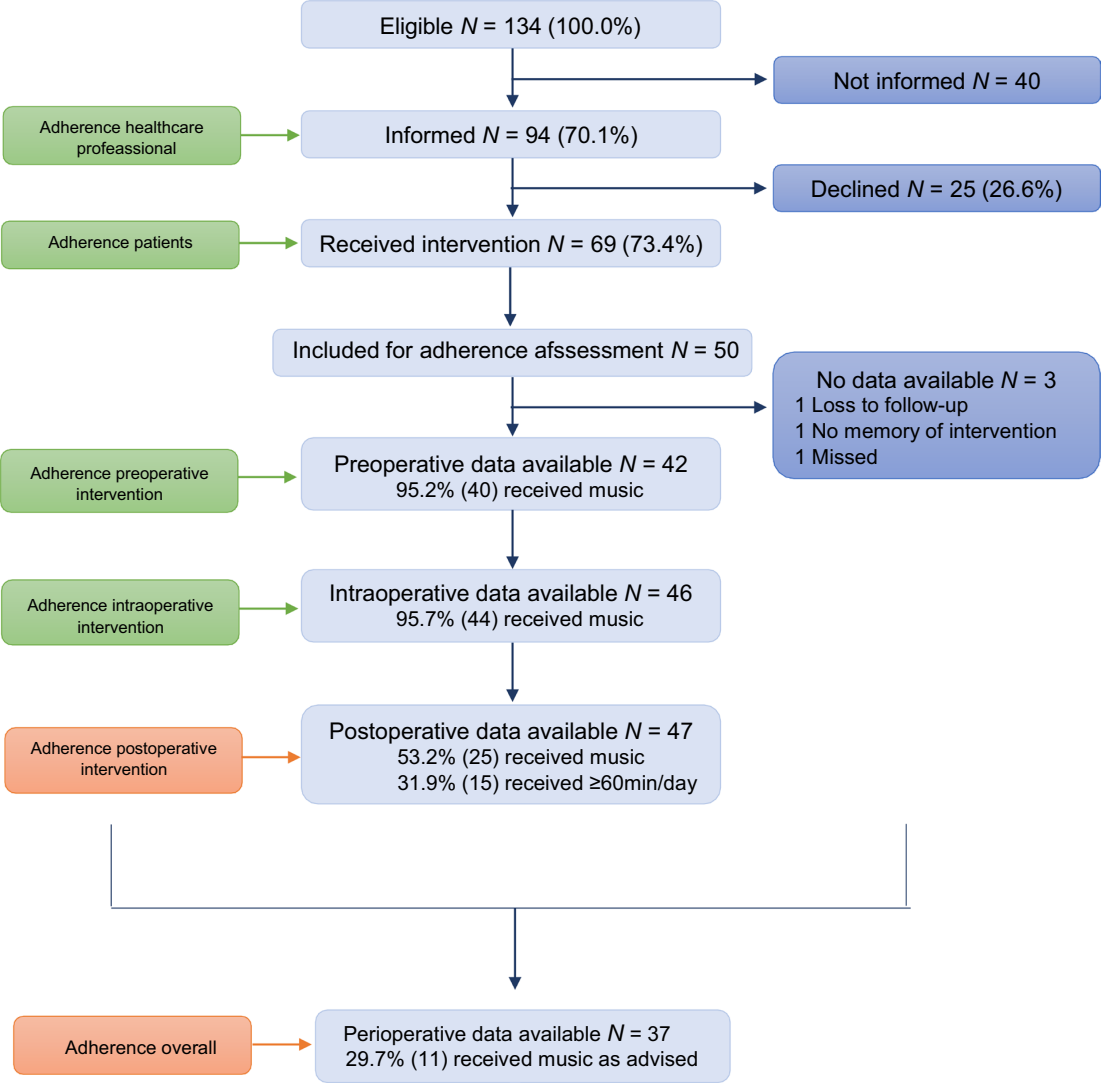
Patient inclusion flowchart and adherence rates to music intervention.

The first patient received music intervention on 9 April 2021. Seventy per cent (94/134) of the eligible patients had been informed about the intervention preoperatively and, eventually, 73.4% (69/94) were willing to receive the intervention (Figure 3). Three patients in the music group were excluded from the analysis: one was lost to follow‐up, one was inadvertently omitted and one had no active recollection of the intervention. Forty‐seven patients were included in the data analysis.

### Effects of implemented music intervention on patient outcomes perioperative anxiety and pain

Postoperative anxiety was significantly lower in the music group (2.0, IQR 0.5–3.0) compared to the control group (3.0, IQR 1.0–5.8), with a *p* value of 0.02. This difference remained significant after correcting for preoperative anxiety (*p* = 0.03). No differences in perioperative pain scores were found between the groups after correcting for sex and age (Table 2). The mixed linear regression analysis did not yield any significant results (*p* = 0.52).

**TABLE 2:** Initial impact of intervention on pain and anxiety.

	*N*	Pre‐implementation	*N*	Post‐implementation	*p* univariate	*p* * multivariate
Intervention as planned						
Pain, mean/median (SD/IQR)						
Day of surgery	42	2.9 (1.4)	11	2.2 (1.1)	0.10	0.43
POD 1	50	2.9 (1.5)	11	2.8 (2.1)	0.81	0.42
POD 2	50	2.2 (1.5–3.3)	11	2.0 (1.6–4.0)	0.54	0.30
POD 3	50	2.3 (1.0–3.8)	11	1.5 (0.3–2.8)	0.20	0.38
POD 4	38	2.2 (0.6–3.0)	8	1.8 (0.8–3.1)	0.86	0.92
POD 5	27	2.0 (0.7–3.3)	6	0.0 (0.0–0.8)	0.02	0.10
POD 6	20	2.4 (1.9–2.4)	2	0.0 (0.0–0.0)	0.06	0.07
POD 7	15	2.0 (0.9–3.0)	3	0.0 (0.0–0.5)	0.07	0.18
Anxiety						
Preoperative	50	4.5 (1.0–7.0)	11	5.0 (2.5–7.5)	0.59	0.06
Postoperative	50	3.0 (1.0–5.8)	11	2.0 (0.5–3.0)	0.26	0.02
Overall						
Pain, mean/median (SD/IQR)						
Day of surgery	42	2.9 (1.4)	50	2.5 (1.2)	0.17	0.43
POD 1	50	2.9 (1.5)	48	2.8 (1.6)	0.68	0.70
POD 2	50	2.2 (1.5–3.3)	49	2.0 (1.0–3.5)	0.90	0.80
POD 3	50	2.3 (1.0–3.8)	48	2.0 (0.9–3.0)	0.56	0.98
POD 4	38	2.2 (0.6–3.0)	35	2.0 (1.0–3.0)	0.86	0.83
POD 5	27	2.0 (0.7–3.3)	22	1.8 (0.5–3.0)	0.96	0.63
POD 6	20	2.2 (1.2)	13	2.1 (2.0)	0.84	0.85
POD 7	15	2.0 (0.9–3.0)	13	1.5 (1.0–2.3)	0.64	0.90
Anxiety						
Preoperative	50	4.5 (1.0–7.0)	50	5.0 (3.0–6.8)	0.72	0.19
Post operative	50	3.0 (1.0–5.8)	50	2.5 (1.0–4.0)	0.45	0.46

Abbreviations: IQR, interquartile range; *N*, number of patients; POD, postoperative day; SD, standard deviation.

* Corrected for sex and age.

### Medication requirement and other outcomes

No significant difference was found in medication requirements before and during surgery (Table 3). However, on both POD 1 and POD 2, more patients in the music group received opioids compared to the control group (36 vs. 27, *p* = 0.047 on POD 1, and 42 vs. 28 on POD 2, *p* = 0.005). No significant difference was found in total dosages of opioids between the groups on POD 1 (*p* = 0.07), but on POD 2 patients in the music group had received a higher dosage of opioids (*p* = 0.03). On POD 1, none of the patients in the music group had received benzodiazepines, whereas five patients in the control group had received benzodiazepines (*p* = 0.04). The groups did not differ in hospital LOS, postoperative LOS, operation duration, 30‐day mortality, 90‐day readmission or complications (Table 2).

**TABLE 3:** Medication requirement.

Medication requirement	Pre‐implementation	Post‐implementation	*p*	*p* *	Dosage^a^		*p*	*p* *
	*N*	*N*			Pre‐implementation	Post‐implementation		
Preoperative								
Naproxen	10	12	0.81	0.68	50.0 (101.0)	60.0 (107.9)	0.63	0.68
Intraoperative								
Epidural	9	8	1.00	0.54	NA	NA	NA	NA
Morphine, mg	45	44	1.00	0.80	1.5 (3.7)	1.3 (3.9)	0.46	0.55
Sufentanil, μg	7	6	1.00	0.39	9.6 (4.6)	9.9 (4.7)	0.77	0.51
Postoperative								
MME, parenteral, mg								
Day of surgery	40	44	0.41	0.10	14.5 (8.3)	11.2 (8.1)	0.70	0.95
POD 1	27	36	0.06	0.047	9.0 (3.5)	9.4 (3.5)	0.07	0.07
POD 2	28	42	0.01	0.005	6.4 (6.7)	9.5 (5.1)	0.01	0.03
POD 3	23	23	1.00	0.71	5.2 (6.2)	5.4 (6.3)	0.68	0.51
POD 4	18	13	0.28	0.27	4.8 (7.7)	3.0 (5.3)	0.28	0.27
POD 5	11	9	0.80	0.82	2.9 (6.3)	1.4 (3.6)	0.52	0.28
POD 6	6	5	1.00	0.67	1.4 (4.7)	1.0 (2.9)	0.68	0.23
POD 7	3	4	1.00	0.65	0.9 (3.4)	1.0 (3.1)	0.70	0.75
NSAIDs								
Day of surgery	16	8	0.10	0.06				
POD 1	10	5	0.26	0.16				
POD 2	12	6	0.19	0.19				
POD 3	3	3	1.00	0.55				
POD 4	2	2	1.00	0.67				
POD 5	2	1	1.00	0.80				
POD 6	0	0	NA	NA				
POD 7	1	0	1.00	1.00				
PCA, days	3.0 (1.3–4.0)	2.0 (2.0–4.0)	0.42	0.66				
Epidural, days	0.0 (0.0–0.0)	0.0 (0.0–0.0)	0.58	0.45				
Benzodiazepines (lorazepam equivalents)								
Day of surgery	5	0	0.07	0.04				
POD 1	4	3	1.00	0.86				
POD 2	5	3	0.71	0.55				
POD 3	3	2	1.00	0.84				
POD 4	3	3	1.00	0.98				
POD 5	2	3	1.00	0.48				
POD 6	1	2	1.00	0.49				
POD 7	1	2	1.00	0.49				
Other secondary outcomes								
Hospital LOS, days	5.5 (4.0–9.0)	4.0 (4.0–6.0)	0.24	0.85				
Postoperative LOS, days	5.0 (4.0–8.0)	4.0 (4.0–6.0)	0.38	0.65				
Operation duration, min	197.5 (163.5–241.3)	171.0 (149.0–241.8)	0.32	0.36				
30‐day mortality	1	0	1.0	1.00				
90‐day re‐admission	3	5	0.71	0.28				
Complication	18	20	0.84	0.57				
Clavien–Dindo grade, number of complications								
0 (no complications)	32	30	0.40					
1	5	8						
2	18	19						
3a	6	13						
3b	1	2						
4a	4	1						
4b	0	0						
5	1	0						

*Note*: Values are reported in mean (SD) or median (IQR). Based on the Shapiro–Wilk test of normality means (SD) or medians (IQR) are reported.

Abbreviations: IQR, interquartile range; LOS, length of stay; MME, morphine milligram equivalents; *N*, number of patients; NSAIDs, non‐steroidal anti‐inflammatory drugs; PCA, patient‐controlled analgesia; POD, postoperative day; SD, standard deviation.

^a^ Means and SDs for dosages were reported but tested with Wilcoxon rank sum test due to non‐normality.

* Corrected for sex and age.

### Process evaluations

During the implementation phase, the initial implementation strategy was adjusted twice. Initially, the nurses at the ward received a list of eligible patients every week. Five months after initiation of the implementation phase, this was altered to an automatic order for music intervention, which was added to the patient's electronic medical file as part of the enhanced recovery after surgery (ERAS) protocol [59].

### Music intervention

Patients listened to their own choice of music, preoperatively, intraoperatively and postoperatively twice daily, 30‐min recorded music sessions via headphones throughout their hospital stay (Figure 2). Forty‐five (95.2% [45/47]) had received the music intervention preoperatively, 45 (95.7% [44/46]) intraoperatively and 15 (31.9% [15/47]) postoperatively for at least 60 min per day. Only 14 (29.7% [14/47]) received the complete music intervention as initially planned (overall adherence; depicted in Figure 3). On average, patients listened to music for 53.8 (standard deviation 103.9) min per day postoperatively. The most chosen music genres were pop (26.1%), classical (21.7%), jazz (10.9%), rock (10.9%) and other (30.4%).

### Evaluation of patient experiences

The complete results can be found in Data S3. Eighty per cent (36/45) of the responding patients stated that the information provision was both timely and sufficient, and they found it understandable. Patients' experiences with the music intervention on the day of surgery ranged from good (37.0%) to very good (39.1%), with a median score of 8.0 (7.0–9.0) on a scale from zero to 10. Likewise, among the patients who also participated in the postoperative setting (53.2%), the experience with the music intervention on the days after surgery was rated as good (38.6%) to very good (15.9%), with a median score of 8.0 (7.0–9.0).

### Healthcare professionals

Sixty‐eight per cent (68/100) of the healthcare professionals involved in the colorectal perioperative process completed the post‐implementation survey. The healthcare professional groups (nurses, specialists and residents) differed significantly from each other regarding age (*p* = 0.005), work experience (*p* < 0.001) and workload (*p* < 0.001), which aligns with findings from the recently published pre‐implementation survey [38]. Complete results are available in Data S3. The pre‐implementation survey identified the most important barriers for implementation as a lack of knowledge about the intervention and an unsupportive professional and culture climate among nurses [38]. The post‐implementation mean knowledge score was 63.6 and was not significantly higher than the pre‐implementation score of 58.3 (Wilcoxon rank sum test; *p* = 0.142). Nevertheless, most of the professionals agreed that the intervention aligns with the current standard practice (pre‐implementation 65.6% vs. post‐implementation 82.8%, Fisher's exact test *p* = 0.03). A significant positive shift (Fisher's exact test *p* < 0.001) was observed in the expected impact the intervention would have on the professionals' daily practice, that is, from ‘low impact’ (pre‐implementation 83.9%, post‐implementation 50.0%) to ‘no impact’ (pre‐implementation 16.1%, post‐implementation 39.7%). Furthermore, 80% of the healthcare professionals expressed their intention to continue offering music interventions because of the positive influence these interventions exert on the patients.

## DISCUSSION AND CONCLUSIONS

The primary goal of the study was to implement recorded music in elective colorectal surgery as standard of care and to assess the adherence to the music and its clinical effects. The conducted tailored strategy resulted in good adherence to music intervention in the preoperative and intraoperative phases and reasonable adherence in the postoperative phase. Seventy‐three per cent of eligible patients (*N* = 69) were willing to receive music intervention. The overall adherence to music intervention, approximately 30%, was exactly as foreseen. Patients highly appreciated the music interventions. The postoperative anxiety scores in the post‐implementation music group were significantly lower than those in the control group (2.0 vs. 3.0, *p* = 0.02). None of the patients in the post‐implementation music group consumed benzodiazepines on the first POD, whereas five patients in the pre‐implementation control group consumed benzodiazepines on the first POD (*p* = 0.04).

The willingness of patients to receive music in the perioperative setting was high in all previously conducted implementation studies, ranging from 68% to 97.1% across studies [31, 33, 53, 54], compared to 73.4% in the present study. This indicates the need for managing surgical patients' anxiety, pain and stress. Nevertheless, approximately one‐fourth of the patients did not wish to receive perioperative music, since not everyone enjoys music. A study on music listening frequency in the United States in 2019 concluded that approximately 8% of the population does not listen to music at all and 11% listens to music less than 1 day per week [55]. Patient satisfaction in all the previously mentioned implementation studies was high, as was the case in this study. Intraoperative music intervention seems to be highly feasible, considering the 81.0% adherence rate in the study by Reudink et al. [53] and 95.7% in this study. Fifty‐three per cent of the patients in this study listened to music in the postoperative setting, while only 29.7% received the complete intervention as planned. This discrepancy may be attributed to the relatively strict assessment of the recommended dose of music throughout the entire hospital admission.

Current literature lacks specifics on the frequency and duration of music intervention necessary to assess dose–effect relationships on patient health outcomes. Fu et al. [17, 56] found that music intervention for 80–120 min daily significantly reduced anxiety and sedative/analgesic needs [17, 57]. Even 60 min daily, as in our study, decreased postoperative anxiety and benzodiazepine use [22, 23, 24, 25, 26]. Thus, offering music intervention on operation or POD 1 is advised to mitigate postoperative anxiety. The significant difference in opioid consumption on POD 1 could suggest that patients in the music group needed relatively lower dosages of opioids than did patients in the control group, since the same dosage of opioids was divided among the greater number of patients in the music group. This difference was also found on POD 2, but on this day opioids consumption in the music group was higher (*p* = 0.03). It could be argued that the higher number of patients in the music group who underwent a laparotomy in the post‐implementation phase (six vs. two in the control group; *p* = 0.14) accounts for this difference in opioid consumption, and that music listening did not influence pain intensity. Further research should investigate the role of opioid consumption. A few studies have considered the placebo effect music might have. A large randomized controlled trial by Chlan et al. [57] compared the effect of music and the use of noise‐cancelling headphones to a control group. They found a significant positive effect of music but not of noise‐cancelling compared to the control group.

This study demonstrated that, even for a readily applicable intervention like music listening, systematic implementation is essential. To maximize the effectiveness of the intervention, adjustments to the initial implementation strategy may be necessary, such as providing more education or extending the implementation period. Our process evaluations were the occasion to make two adjustments that might improve the postoperative intervention adherence, which led to a slight increase.

## STRENGTHS AND LIMITATIONS

This pioneering study introduced perioperative music intervention into standard elective care throughout a hospital stay, aided by an implementation expert (EI) and a widely used framework. Its protocol offers a blueprint for integrating music interventions in various settings. Multilevel strategies, as outlined here, are more effective than single approaches [48]. The design of the study, a pre‐ and post‐implementation pilot study with a limited sample size in a single centre, has limited its ability to detect effects of music on clinical outcomes such as pain, medication use and so forth. Nevertheless, the intervention appeared to have a positive impact on patients' anxiety and their consumption of benzodiazepines and opioids. However, retrospective assessment of preoperative anxiety levels may introduce recall bias.

### Future research perspectives

The systematic and efficient implementation of effective interventions remains a challenge within the current healthcare system. ERAS protocols have been demonstrated to shorten hospital length of stay and improve outcomes of patients undergoing colorectal surgery, to which music intervention could well be added [58, 59]. However, even years after accumulating evidence supporting the use of the ERAS protocol was acquired, achieving effective implementation still demands great effort [58]. Implementation studies have shown that successful implementation is context and setting dependent [31], which implies that the same implementation strategy or intervention can be effective in one setting but not in another. Other possible barriers might include logistics such as availability of music, licences, facilities like internet or headphones, cultural/religious barriers which inhibit the use of music and financial (especially in less developed countries) or the clinical relevance, which might be one of the most important factors. These factors, and some others that could be detected when systematic assessment was carried out as described in the previously published paper of the IMPROVE study [38], have influence on the feasibility. As mentioned before, in this monocentre pilot implementation study it was not possible to study effectiveness of the intervention. We therefore advise large‐scale multicentre studies implementing music and assessing the effect of music in a randomized setting with three arms including a music, control and noise‐cancelling group. Fast and effective implementation becomes achievable once evidence of feasibility is obtained. Unfortunately, it might take some time before large‐scale studies are carried out, more effective approaches are found and results are presented. Since music has shown positive clinical relevant effects, on the basis of which a guideline for the use of perioperative music has been developed in the Netherlands, and does not show any harmful effects, it can already be safely considered as part of the standard care.

## CONCLUSIONS

This pilot implementation study showed that music can be successfully integrated into preoperative and intraoperative phases, making it feasible for elective perioperative colorectal care. The implementation of postoperative music intervention additionally necessitates tailored, multilevel implementation strategies, and a subsequent process evaluation. The implementation of music might have a positive impact, reducing patients' postoperative subjective anxiety as well as the consumption of benzodiazepines and opioids. Both patients and healthcare professionals expressed enthusiasm and reported good experiences. Music is easily applicable, presents no known risks and can be safely considered as part of the standard perioperative care.

## AUTHOR CONTRIBUTIONS

**Ellaha Kakar:** Formal analysis; writing – original draft; investigation. **Oddeke van Ruler:** Writing – review and editing; methodology; supervision. **Bas Hoogteijling:** Supervision; writing – review and editing; methodology. **Eelco J. R. de Graaf:** Supervision; methodology; writing – review and editing. **Erwin Ista:** Supervision; methodology; writing – original draft; writing – review and editing. **Johan F. Lange:** Supervision; conceptualization; writing – original draft; writing – review and editing; methodology. **Johannes Jeekel:** Supervision; conceptualization; writing – original draft; funding acquisition; writing – review and editing; methodology. **Markus Klimek:** Supervision; conceptualization; writing – original draft; funding acquisition; writing – review and editing.

## FUNDING INFORMATION

Erasmus MC Foundation, Music as Medicine; Van Cappellen Stichting, Stichting Theia.

## CONFLICT OF INTEREST STATEMENT

The authors declare no conflict of interest.

## ETHICS STATEMENT

The study protocol was approved by to the Medical Ethics Review Board of the Erasmus University Medical Centre (registration number MEC‐2019‐0563).

## CLINICAL TRIAL REGISTRATION

Nederlands Trial Register, Trial number NL8071.

## PREREGISTRATION OF STUDY OR ANALYSIS PLAN DISCLOSURE

No preregistration exists for the studies reported in this article.

## Supporting information


Data S1.

## Data Availability

Due to the sensitive nature of the data collected for this study, requests to access the dataset from qualified researchers trained in human subject confidentiality protocols may be sent to the corresponding author of this study.

## References

[1] De Oliveira GS Jr , Holl JL , McCarthy RJ , Butt ZA , Nouriel J , McCaffery K , et al. Overestimation of mortality risk and preoperative anxiety in patients undergoing elective general surgery procedures: a propensity matched analysis. Int J Surg. 2014;12(12):1473–1477. 25463769 10.1016/j.ijsu.2014.11.016

[2] Robleda G , Sillero‐Sillero A , Puig T , Gich I , Baños JE . Influence of preoperative emotional state on postoperative pain following orthopedic and trauma surgery. Rev Lat Am Enfermagem. 2014;22(5):785–791. 25493674 10.1590/0104-1169.0118.2481 PMC4292684

[3] Maranets I , Kain ZN . Preoperative anxiety and intraoperative anesthetic requirements. Anesth Analg. 1999;89(6):1346–1351. 10589606 10.1097/00000539-199912000-00003

[4] Kindler CH , Harms C , Amsler F , Ihde‐Scholl T , Scheidegger D . The visual analog scale allows effective measurement of preoperative anxiety and detection of patients' anesthetic concerns. Anesth Analg. 2000;90(3):706–712. 10702461 10.1097/00000539-200003000-00036

[5] Gras S , Servin F , Bedairia E , Montravers P , Desmonts JM , Longrois D , et al. The effect of preoperative heart rate and anxiety on the propofol dose required for loss of consciousness. Anesth Analg. 2010;110(1):89–93. 19910628 10.1213/ANE.0b013e3181c5bd11

[6] Mijderwijk H , van Beek S , Klimek M , Duivenvoorden HJ , Grüne F , Stolker RJ . Lorazepam does not improve the quality of recovery in day‐case surgery patients: a randomised placebo‐controlled clinical trial. Eur J Anaesthesiol. 2013;30(12):743–751. 23635914 10.1097/EJA.0b013e328361d395

[7] Ip HY , Abrishami A , Peng PW , Wong J , Chung F . Predictors of postoperative pain and analgesic consumption: a qualitative systematic review. Anesthesiology. 2009;111(3):657–677. 19672167 10.1097/ALN.0b013e3181aae87a

[8] Sommer M , de Rijke JM , van Kleef M , Kessels AGH , Peters ML , Geurts JWJM , et al. The prevalence of postoperative pain in a sample of 1490 surgical inpatients. Eur J Anaesthesiol. 2008;25(4):267–274. 18053314 10.1017/S0265021507003031

[9] Cao X , Elvir‐Lazo OL , White PF , Yumul R , Tang J . An update on pain management for elderly patients undergoing ambulatory surgery. Curr Opin Anaesthesiol. 2016;29(6):674–682. 27820738 10.1097/ACO.0000000000000396

[10] Gan TJ . Poorly controlled postoperative pain: prevalence, consequences, and prevention. J Pain Res. 2017;10:2287–2298. 29026331 10.2147/JPR.S144066 PMC5626380

[11] Kumar A , Dubey PK , Ranjan A . Assessment of anxiety in surgical patients: an observational study. Anesth Essays Res. 2019;13(3):503–508. 31602069 10.4103/aer.AER_59_19 PMC6775825

[12] Neuendorf R , Harding A , Stello N , Hanes D , Wahbeh H . Depression and anxiety in patients with inflammatory bowel disease: a systematic review. J Psychosom Res. 2016;87:70–80. 27411754 10.1016/j.jpsychores.2016.06.001

[13] Kappelman MD , Long MD , Martin C , DeWalt DA , Kinneer PM , Chen W , et al. Evaluation of the patient‐reported outcomes measurement information system in a large cohort of patients with inflammatory bowel diseases. Clin Gastroenterol Hepatol. 2014;12(8):1315–1323.e2. 24183956 10.1016/j.cgh.2013.10.019 PMC4361943

[14] Byrne G , Rosenfeld G , Leung Y , Qian H , Raudzus J , Nunez C , et al. Prevalence of anxiety and depression in patients with inflammatory bowel disease. Can J Gastroenterol Hepatol. 2017;2017:6496727. 29181373 10.1155/2017/6496727 PMC5664260

[15] Shoar S , Naderan M , Aghajani M , Sahimi‐Izadian E , Hosseini‐Araghi N , Khorgami Z . Prevalence and determinants of depression and anxiety symptoms in surgical patients. Oman Med J. 2016;31(3):176–181. 27162587 10.5001/omj.2016.35 PMC4852078

[16] Ghoneim MM , O'Hara MW . Depression and postoperative complications: an overview. BMC Surg. 2016;16:5. 26830195 10.1186/s12893-016-0120-y PMC4736276

[17] Fu VX , Oomens P , Klimek M , Verhofstad MHJ , Jeekel J . The effect of perioperative music on medication requirement and hospital length of stay: a meta‐analysis. Ann Surg. 2019;272:961–972. 10.1097/SLA.0000000000003506 PMC7668322 31356272

[18] Fu VX , Oomens P , Sneiders D , van den Berg SAA , Feelders RA , Wijnhoven BPL , et al. The effect of perioperative music on the stress response to surgery: a meta‐analysis. J Surg Res. 2019;244:444–455. 31326711 10.1016/j.jss.2019.06.052

[19] Kuhlmann AYR , van Rosmalen J , Staals LM , Keyzer‐Dekker CMG , Dogger J , de Leeuw TG , et al. Music interventions in pediatric surgery (the music under surgery in children study): a randomized clinical trial. Anesth Analg. 2019;130(4):991–1001. 10.1213/ANE.0000000000003983 30633058

[20] Kakar E , Billar RJ , van Rosmalen J , Klimek M , Takkenberg JJM , Jeekel J . Music intervention to relieve anxiety and pain in adults undergoing cardiac surgery: a systematic review and meta‐analysis. Open Heart. 2021;8(1):e001474. 33495383 10.1136/openhrt-2020-001474 PMC7839877

[21] Kuhlmann AYR , de Rooij A , Kroese LF , van Dijk M , Hunink MGM , Jeekel J . Meta‐analysis evaluating music interventions for anxiety and pain in surgery. Br J Surg. 2018;105(7):773–783. 29665028 10.1002/bjs.10853 PMC6175460

[22] Carroll JK , Cullinan E , Clarke L , Davis NF . The role of anxiolytic premedication in reducing preoperative anxiety. Br J Nurs. 2012;21(8):479–483. 22585076 10.12968/bjon.2012.21.8.479

[23] Buscemi N , Vandermeer B , Friesen C , Bialy L , Tubman M , Ospina M , et al. The efficacy and safety of drug treatments for chronic insomnia in adults: a meta‐analysis of RCTs. J Gen Intern Med. 2007;22(9):1335–1350. 17619935 10.1007/s11606-007-0251-z PMC2219774

[24] Holbrook AM , Crowther R , Lotter A , Cheng C , King D . Meta‐analysis of benzodiazepine use in the treatment of insomnia. CMAJ. 2000;162(2):225–233. 10674059 PMC1232276

[25] National Institutes of Health . National Institutes of Health State of the Science Conference statement on manifestations and management of chronic insomnia in adults, June 13–15, 2005. Sleep. 2005;28(9):1049–1057. 16268373 10.1093/sleep/28.9.1049

[26] Nowell PD , Mazumdar S , Buysse DJ , Dew MA , Reynolds CF 3rd , Kupfer DJ . Benzodiazepines and zolpidem for chronic insomnia: a meta‐analysis of treatment efficacy. JAMA. 1997;278(24):2170–2177. 9417012

[27] Zaal IJ , Devlin JW , Hazelbag M , Klein Klouwenberg PMC , van der Kooi AW , Ong DSY , et al. Benzodiazepine‐associated delirium in critically ill adults. Intensive Care Med. 2015;41(12):2130–2137. 26404392 10.1007/s00134-015-4063-z

[28] van Beek S , Kroon J , Rijs K , Mijderwijk HJ , Klimek M , Stolker RJ . The effect of midazolam as premedication on the quality of postoperative recovery after laparotomy: a randomized clinical trial. Can J Anaesth. 2020;67(1):32–41. 31576513 10.1007/s12630-019-01494-6

[29] Brummett CM , Waljee JF , Goesling J , Moser S , Lin P , Englesbe MJ , et al. New persistent opioid use after minor and major surgical procedures in US adults. JAMA Surg. 2017;152(6):e170504. 28403427 10.1001/jamasurg.2017.0504 PMC7050825

[30] Halfens R , Cox K , Kuppen‐Van MA . Effect of the use of sleep medication in Dutch hospitals on the use of sleep medication at home. J Adv Nurs. 1994;19(1):66–70. 8138632 10.1111/j.1365-2648.1994.tb01052.x

[31] Carter JE , Pyati S , Kanach FA , Maxwell AMW , Belden CM , Shea CM , et al. Implementation of perioperative music using the consolidated framework for implementation research. Anesth Analg. 2018;127(3):623–631. 29905616 10.1213/ANE.0000000000003565

[32] Hebert CA , Hancock K , McConnell ES . Implementation of individualized music in long‐term care: application of the PARiHS framework. J Gerontol Nurs. 2018;44(8):29–38. 10.3928/00989134-20180626-01 30059137

[33] Sharda N , Mattoon E , Matters L , Prewitt J , McDonald S , Sloane R , et al. Bach to the basics: implementation and impact of a postoperative, inpatient personalized music program for older adults. J Perianesth Nurs. 2019;34(2):347–353. 30205935 10.1016/j.jopan.2018.05.006

[34] Kakar E , Ista E , Klimek M , Jeekel J . Implementation of music in the perioperative standard care of colorectal surgery: study protocol of the IMPROVE Study. BMJ Open. 2021;11(10):e051878. 10.1136/bmjopen-2021-051878 PMC8557300 34711596

[35] Proctor E , Silmere H , Raghavan R , Hovmand P , Aarons G , Bunger A , et al. Outcomes for implementation research: conceptual distinctions, measurement challenges, and research agenda. Admin Pol Ment Health. 2011;38(2):65–76. 10.1007/s10488-010-0319-7 PMC3068522 20957426

[36] Damschroder LJ , Aron DC , Keith RE , Kirsh SR , Alexander JA , Lowery JC . Fostering implementation of health services research findings into practice: a consolidated framework for advancing implementation science. Implement Sci. 2009;4:50. 19664226 10.1186/1748-5908-4-50 PMC2736161

[37] Birken SA , Powell BJ , Shea CM , Haines ER , Alexis Kirk M , Leeman J , et al. Criteria for selecting implementation science theories and frameworks: results from an international survey. Implement Sci. 2017;12(1):124. 29084566 10.1186/s13012-017-0656-y PMC5663064

[38] Kakar E , van Ruler O , van Straten B , Hoogteijling B , de Graaf EJR , Ista E , et al. Implementation of music in colorectal perioperative standard care—barriers and facilitators among patients and healthcare professionals. Colorectal Dis. 2022;24:868–875. 35194930 10.1111/codi.16102 PMC9544166

[39] Dindo D , Demartines N , Clavien PA . Classification of surgical complications: a new proposal with evaluation in a cohort of 6336 patients and results of a survey. Ann Surg. 2004;240(2):205–213. 15273542 10.1097/01.sla.0000133083.54934.ae PMC1360123

[40] Clavien PA , Barkun J , de Oliveira ML , Vauthey JN , Dindo D , Schulick RD , et al. The Clavien–Dindo classification of surgical complications: five‐year experience. Ann Surg. 2009;250(2):187–196. 19638912 10.1097/SLA.0b013e3181b13ca2

[41] Howard P , Twycross R , Shuster J , Mihalyo M , Wilcock A . Benzodiazepines. J Pain Symptom Manag. 2014;47(5):955–964. 10.1016/j.jpainsymman.2014.03.001 24681184

[42] Girard TD , Exline MC , Carson SS , Hough CL , Rock P , Gong MN , et al. Haloperidol and ziprasidone for treatment of delirium in critical illness. N Engl J Med. 2018;379(26):2506–2516. 30346242 10.1056/NEJMoa1808217 PMC6364999

[43] VInkers CHTJ , Luykx JJ , Vis R . Kiezen voor de juiste benzodiazepine: werkingsmechanisme en farmacokinetiek. Ned Tijdschr Geneeskd. 1987;131(16):651–655. 22929751

[44] Pinnock H , Barwick M , Carpenter CR , Eldridge S , Grandes G , Griffiths CJ , et al. Standards for Reporting Implementation Studies (StaRI): explanation and elaboration document. BMJ Open. 2017;7(4):e013318. 10.1136/bmjopen-2016-013318 PMC5387970 28373250

[45] Wensing MGR , Grimshaw J . Improving patient care; the implementation of change in clinical practice. 3rd ed. New Jersey: Wiley Blackwell; 2020.

[46] Powell BJ , Waltz TJ , Chinman MJ , Damschroder LJ , Smith JL , Matthieu MM , et al. A refined compilation of implementation strategies: results from the Expert Recommendations for Implementing Change (ERIC) project. Implement Sci. 2015;10:21. 25889199 10.1186/s13012-015-0209-1 PMC4328074

[47] Proctor EK , Powell BJ , McMillen JC . Implementation strategies: recommendations for specifying and reporting. Implement Sci. 2013;8:139. 24289295 10.1186/1748-5908-8-139 PMC3882890

[48] Baker R , Camosso‐Stefinovic J , Gillies C , Shaw EJ , Cheater F , Flottorp S , et al. Tailored interventions to overcome identified barriers to change: effects on professional practice and health care outcomes. Cochrane Database Syst Rev. 2010;1(3):CD005470. 10.1002/14651858.CD005470.pub2 PMC4164371 20238340

[49] Kaminski JP , Pai A , Ailabouni L , Park JJ , Marecik SJ , Prasad LM , et al. Role of epidural and patient‐controlled analgesia in site‐specific laparoscopic colorectal surgery. JSLS. 2014;18(4):e2014.00207. 10.4293/JSLS.2014.00207 PMC4234047 25419110

[50] Mouawad NJ , Leichtle SW , Kaoutzanis C , Welch K , Winter S , Lampman R , et al. Pain control with continuous infusion preperitoneal wound catheters versus continuous epidural analgesia in colon and rectal surgery: a randomized controlled trial. Am J Surg. 2018;215(4):570–576. 28688514 10.1016/j.amjsurg.2017.06.031

[51] Kelly AM . The minimum clinically significant difference in visual analogue scale pain score does not differ with severity of pain. Emerg Med J. 2001;18(3):205–207. 11354213 10.1136/emj.18.3.205 PMC1725574

[52] Chakraborty A . Bounded influence function based inference in joint modelling of ordinal partial linear model and accelerated failure time model. Stat Methods Med Res. 2016;25(6):2714–2732. 24770852 10.1177/0962280214531570

[53] Reudink M , Fu VX , Mackenbach KTR , Jeekel J , Slooter GD , Dias EM . Implementation of perioperative music in day care surgery. Acta Chir Belg. 2021;123:1–9. 10.1080/00015458.2021.1988232 34641770

[54] Xu X , Sun BL , Huang F , Chia HLA , Sultana R , Teo A , et al. The impact of music on patient satisfaction, anxiety, and depression in patients undergoing gynecologic surgery. J Perianesth Nurs. 2021;36(2):122–127. 33388222 10.1016/j.jopan.2020.08.014

[55] Statista . Music listening frequency in the United States as of June 2019, by age group. 2019. https://www.statista.com/statistics/972958/music‐listening‐frequency‐habits‐usa/#:~:text=According%20to%20the%20most%20recent,to%20three%20times%20per%20week

[56] Chlan L , Heiderscheit A . A tool for music preference assessment in critically ill patients receiving mechanical ventilatory support. Music Ther Perspect. 2009;27(1):42–47. 24489432 10.1093/mtp/27.1.42 PMC3905330

[57] Chlan LL , Weinert CR , Heiderscheit A , Tracy MF , Skaar DJ , Guttormson JL , et al. Effects of patient‐directed music intervention on anxiety and sedative exposure in critically ill patients receiving mechanical ventilatory support: a randomized clinical trial. JAMA. 2013;309(22):2335–2344. 23689789 10.1001/jama.2013.5670 PMC3683448

[58] Byrnes A , Young A , Mudge A , Banks M , Clark D , Bauer J . Prospective application of an implementation framework to improve postoperative nutrition care processes: evaluation of a mixed methods implementation study. Nutr Diet. 2018;75(4):353–362. 30151938 10.1111/1747-0080.12464

[59] Cavallaro P , Bordeianou L . Implementation of an ERAS pathway in colorectal surgery. Clin Colon Rectal Surg. 2019;32(2):102–108. 30833858 10.1055/s-0038-1676474 PMC6395097

