# Supplementary material for: Implementation of music in the perioperative standard care of colorectal surgery (IMPROVE study)
Source: Colorectal Dis. 2024 Oct 9;26(12):2080–91. doi: 10.1111/codi.17200 (PMC11649877; doi:10.1111/codi.17200)
Supplement: Supplementary file 1 — Data S1. [file CODI-26-2080-s001.docx]

Supplementary files

supplemental file 1. Patient process evaluation questionnaire

Anxiety

Question 1a. How anxious were you before surgery?

Not anxious at all Very anxious

□ □ □ □ □ □ □ □ □ □ □

0 1 2 3 4 5 6 7 8 9 10

Question 1b. How anxious were you after surgery?

Not anxious at all Very anxious

□ □ □ □ □ □ □ □ □ □ □

0 1 2 3 4 5 6 7 8 9 10

Experience with the music intervention

Question 1. When were you informed regarding the use of music before, during, and after surgery?

1. When also the information regarding anaesthesia was given.
2. When also the information regarding surgery was given.
3. On the day of surgery.
4. Otherwise: …………………………………………………

Question 2. When and for how long did you listen to music during your hospital admission?

1. Days before the surgery: : □ Yes □ No

Which days and how long per day: …………

1. On the day of surgery: □ Yes □ No
2. During surgery: □ Yes □ No
3. After surgery: □ Yes □ No

Which days and how long per day: …………

Question 3. How did you experience listening to music of your preference before and/or after surgery on the day of surgery?

1. Very good
2. Good
3. Neutral
4. Bad
5. Very bad

How would you score this experience on a scale from zero to ten? Answer: …………….

Question 4. How did you experience listening to music of your preference before and/or after surgery in the nursing department?

1. Very good
2. Good
3. Neutral
4. Bad
5. Very bad

How would you score this experience on a scale from zero to ten? Answer: …………….

Question 5. How satisfied were you with the music equipment?

1. Very satisfied
2. Satisfied
3. Neutral
4. Unsatisfied
5. Very unsatisfied

How would you score this satisfaction on a scale from zero to ten? Answer: …………….

Question 6. To what type of music did you listen during your admission?

󠇎 Classical 󠇎 Dutch 󠇎 New Age

󠇎 Jazz 󠇎 Rhythm and Blues (R&B) 󠇎 Alternative Rock

󠇎 Rock 󠇎 Reggae 󠇎 Heavy Metal

󠇎 Religious 󠇎 Country 󠇎 World music

󠇎 Hip Hop 󠇎 Rap 󠇎 Pop

󠇎 Music form the years: ………………………………………………………………..

󠇎 Other: …………………………………………………………………………..……...

Question 7. Would you listen to music again during a next operation?

1. Yes, because;

1. I was informed properly regarding the music

2. I could choose my own music

3. I love music

4. I felt better because of the music

5. Other reason: ……………………………………………………………….

1. No, because;
2. I was not informed sufficiently
3. It cost me too much effort
4. I did not feel well with the music
5. I did not see the added value of the music

Question 8. Please tick what applies to you.

1. The information about music before, during and after the operation was given on time.

󠇎 Agree

󠇎 Disagree, please write down what you think could be improved…………………

1. The information about music before, during and after the operation was sufficient, clear and understandable.

󠇎 Agree

󠇎 Disagree, please write down what you think could be improved…………………

Question 9. If applicable, please provide points for improvement with regard to how music in the IJsselland Hospital is applied.

a. Points for improvement regarding information provision: ……………………………………...

b. Points for improvement regarding music equipment: …………………………………………..

c. Other points for improvement:……………………………………………………………………..

supplemental file 2. Implementation strategy

| **Strategy** | **Definition** | **Specification** |
| --- | --- | --- |
| **Intervention** | | |
| Intervention application | Music should be provided twice a day, preferably in the morning after awakening and evening before bedtime, during a minimum of 30 minutes during the entire admission. Music intervention should be initiated at the nursing ward and not halted untill the patient is back to the nursing ward postoperatively. The music will not interfere during communication with the patient. If needed the headphone can be removed briefly for communication with the patient, but if communication is possible with the patient with the headphone on, this is preferred. Figure 1 gives a brief visualization of the perioperative music intervention process. | **Preoperative**  **Actor**: ward nurses  **Action**: apply music intervention preoperatively when patient is ordered for surgery.  **Target**: implementation process  **Temporality**: executing  **Dose**: preoperative  **Outcome** **affected**: adherence, penetrations  **Intraoperative**  **Actor**: surgeons, anaesthesiologist, nurse anaesthetists  **Action**: check that the music intervention is applied pre-, intra-, and postoperatively.  **Target**: implementation process  **Temporality**: executing  **Dose**: pre-, intra-, and postoperative for every patient  **Outcome** **affected**: adherence, penetrations  **Postoperative**  **Actor**: ward nurses  **Action**: apply/provide/remind patient music intervention postoperatively  **Target**: implementation process  **Temporality**: executing  **Dose**: postoperatively, two times per day, morning and evening, at least 30 minutes per moment, every day  **Outcome** **affected**: adherence, penetrations |
| Equipment | Tablet, over-the-ear headphones, Spotify | **Actor**: research team and MSupport  **Action**: supply music equipment  **Target**: implementation costs  **Temporality**: executing  **Dose**: once  **Outcome** **affected**: costs |
| Music choice | Music choice will be based on the preference of the patient. Music lists will be available based on genre. The patients are also allowed to choose their preferred music freely in the Spotify environment. | **Actor**: patients and nurses  **Action**: choose music list  **Target**: implementation process  **Temporality**: executing  **Dose**: every preoperative contact with patient  **Outcome** **affected**: adherence, penetrations |
| Music volume | Volume will be set at a level which is comfortable for the patient and which does not affect communication between patient and caregiver on the day of the operation. | **Actor**: patients and nurses  **Action**: set the volume  **Target**: implementation process  **Temporality**: executing  **Dose**: once per patient preoperatively  **Outcome** **affected**: adherence |
| **General** | | |
| Use an implementation advisor | Seek guidance from experts in implementation | **Actor**: research team  **Action**: guidance by implementation expert  **Target**: process of implementation  **Temporality**: engaging  **Dose**: regularly during entire implementation process  **Outcome** **affected**: sustainability |
| Provide local technical assistance | Develop and use a system to deliver technical assistance focused on implementation issues using local personnel | **Actor**: research team and MSupport  **Action**: provide help in technical problems with music equipment.  **Target**: intervention characteristics  **Temporality**: executing  **Dose**: ongoing  **Outcome affected**: acceptability, feasibility, adherence, sustainability |
| Purposely reexamine the implementation | Monitor progress and adjust clinical practices and implementation strategies to continuously improve the quality of care | **Actor**: research team, dedicated team  **Action**: surveys/interviews  **Target**: process of implementation  **Temporality**: reflecting/evaluating  **Dose**: monthly  **Outcome affected**: penetration |
| Organize clinician implementation team meetings | Develop and support teams of clinicians who are implementing the innovation and give them protected time to reflect on the implementation effort, share lessons learned, and support one another’s learning | **Actor**: research team, dedicated team  **Action**: team meetings  **Target**: process of implementation  **Temporality**: reflecting/evaluating  **Dose**: when found necessary after monthly meeting/contact with dedicated team  **Outcome** **affected**: adherence, penetration, sustainability |
| Provide ongoing consultation | Provide ongoing consultation with one or more experts in the strategies used to support implementing the innovation | **Actor**: research team  **Action**: provide consultation  **Target**: process of implementation  **Temporality**: executing  **Dose**: ongoing  **Outcome affected**: acceptability, intention to try, adherence, sustainability |
| **Stakeholder (caregivers)** | | |
| Inform local opinion leaders | Inform providers identified by colleagues as opinion leaders or “educationally influential” about the clinical innovation in the hope that they will influence colleagues to adopt it | **Actor**: research team  **Action**: oral presentations  **Target**: knowledge and belief  **Temporality**: planning  **Dose**: twice at initiation of the study  **Outcome** **affected**: knowledge and belief |
| Conduct educational meetings | Hold meetings targeted toward different stakeholder groups (e.g., providers, administrators, other organizational stakeholders, and community, patient/consumer, and family stakeholders) to teach them about the clinical innovation | **Actor**: research team  **Action**: oral presentations  **Target**: knowledge and belief  **Temporality**: executing  **Dose**: twice at initiation of the study  **Outcome** **affected**: knowledge and belief |
| Develop educational materials | Develop and format manuals, toolkits, and other supporting materials in ways that make it easier for stakeholders to learn about the innovation and for clinicians to learn how to deliver the clinical innovation | **Actor**: research team, dedicated team  **Action**: presentations (video), posters, folders, e-mail, newsletters  **Target**: self-efficacy, process of implementation  **Temporality**: planning  **Dose**: at initiation of the implementation phase and based on input at reflection/evaluation  **Outcome** **affected**: intention to try, adherence, sustainability |
| Distribute educational materials | Distribute educational materials (including guidelines, manuals, and toolkits) in person, by mail, and/or electronically | **Actor**: research team, dedicated team  **Action**: posters, folders, e-mail, newsletter  **Target**: self-efficacy, process of implementation  **Temporality**: planning and executing  **Dose**: at initiation of the implementation phase and based on input at reflection/evaluation  **Outcome** **affected**: adherence, sustainability, penetration |
| Facilitate relay of clinical data to providers | Provide as close to real-time data as possible about key measures of process/outcomes using integrated modes/channels of communication in a way that promotes use of the targeted innovation | **Actor**: research team  **Action**: inform stakeholders regarding adherence to the intervention through e-mail, newsletter  **Target**: relative advantage of the intervention  **Temporality**: reflecting/evaluating  **Dose**: during three months after initiation of implementation phase  **Outcome affected**: relevance, adherence, sustainability |
| Remind caregivers | Develop reminder systems designed to help caregivers to recall information and/or prompt them to use the clinical innovation | **Actor**: research team  **Action**:  **posters**, folders  **Target**: stakeholders  **Temporality**: executing  **Dose**: daily  **Outcome** **affected**: adherence |
| Purposely reexamine the implementation | Monitor progress and adjust clinical practices and implementation strategies to continuously improve the quality of care | **Actor**: research team  **Action**: surveys/interviews  **Target**: process of implementation  **Temporality**: reflecting/evaluating  **Dose**: monthly  **Outcome affected**: penetration |
| Use an implementation advisor | Seek guidance from experts in implementation | **Actor**: research team  **Action**: guidance by implementation expert  **Target**: process of implementation  **Temporality**: engaging  **Dose**: continuously  **Outcome** **affected**: sustainability |
| Promote adaptability | Identify the ways a clinical innovation can be tailored to meet local needs and clarify which elements of the innovation must be maintained to preserve adherence | **Actor**: research team, dedicated team  **Action**: add to educational material  **Target**: intervention characteristics  **Temporality**: planning  **Dose**: monthly  **Outcome** **affected**: acceptability, intention to try, feasibility, adherence, sustainability |
| **Stakeholder (patients)** | | |
| Develop educational materials | Develop and format manuals, toolkits, and other supporting materials in ways that make it easier for stakeholders to learn about the innovation | **Actor**: dedicated team, research team  **Action**: information folders, manual for verbal informing  **Target**: knowledge and belief  **Temporality**: planning  **Dose**: every preoperative contact with patient  **Outcome** **affected**: acceptability and adherence |
| Distribute educational materials | Distribute educational materials (including guidelines, manuals, and toolkits) in person, by mail, and/or electronically | **Actor**: dedicated team, research team  **Action**: add information/folders to existing information packages/application. Verbal informing during outpatient clinic visits.  **Target**: knowledge and belief  **Temporality**: executing  **Dose**: every preoperative contact with patient.  **Outcome** **affected**: acceptability and adherence |
| Prepare patients/consumers to be active participants | Prepare patients/consumers to be active in their care, to ask questions, and specifically to inquire about care guidelines, the evidence behind clinical decisions, or about available evidence-supported treatments | **Actor**: research team  **Action**: inform through educational material/outpatient clinic regarding own application of the intervention  **Target**: self-efficacy  **Temporality**: engaging  **Dose**: preoperative in educational material,  **Outcome** **affected**: acceptability, intention to try, and adherence |
| Obtain and use patients/consumers and family feedback | Develop strategies to increase patient/consumer and family feedback on the implementation effort | **Actor**: research team  **Action**: surveys/interviews  **Target**: process of implementation (reflecting/evaluating)  **Temporality**: reflecting/evaluating  **Dose**: once per two weeks  **Outcome** **affected**: adaptation, penetration, sustainability, feasibility |

supplemental file 3. Patient process evaluation

|  | N |  | N | % | Score |
| --- | --- | --- | --- | --- | --- |
| Information provided moment | 44 | Outpatient visit | 25 | 56.8 | NA |
|  |  | Day of surgery | 16 | 36.4 |  |
|  |  | Folder | 3 | 6.8 |  |
| Information provision on time | 45 | Agree | 36 | 80.0 | NA |
| Information proper | 45 | Agree | 37 | 82.2 | NA |
| Experience day of surgery | 46 | Very good | 18 | 39.1 | 8.0 (7.0-9.0) |
|  |  | Good | 17 | 37.0 |  |
|  |  | Neutral | 11 | 23.9 |  |
| Experience days after surgery | 44 | Very good | 7 | 15.9 | 8.0 (7.0-9.0) |
|  |  | Good | 17 | 38.6 |  |
|  |  | Neutral | 1 | 2.2 |  |
|  |  | Did not receive music | 19 | 43.2 |  |
| Music equipment satisfaction | 45 | NA | NA | NA | 8.0 (7.0-8.3) |
| Music next operation | 45 | Yes | 42 | 93.3 | NA |
| Music next operation reason | 46 | Relaxation/ rest/peace | 23 | 50.0 | NA |
|  |  | Distraction | 7 | 15.2 |  |
|  |  | Other | 16 | 34.8 |  |
| Type of music | 46 | Pop | 12 | 26.1 | NA |
|  |  | Classic | 10 | 21.7 |  |
|  |  | Jazz | 5 | 10.9 |  |
|  |  | Rock | 5 | 10.9 |  |
|  |  | Other | 14 | 30.4 |  |
| N; number of patients, NA; not applicable. | | | | | |
